# The Composite Effect Is Face-Specific in Young but Not Older Adults

**DOI:** 10.3389/fnagi.2016.00187

**Published:** 2016-08-05

**Authors:** Günter Meinhardt, Malte Persike, Bozana Meinhardt-Injac

**Affiliations:** Department of Psychology, Johannes Gutenberg University MainzMainz, Germany

**Keywords:** age-related decline, holistic face perception, composite effect, interference, attentional control

## Abstract

In studying holistic face processing across the life-span there are only few attempts to separate face-specific from general aging effects. Here we used the complete design of the composite paradigm (Cheung et al., 2008) with faces and novel non-face control objects (watches) to investigate composite effects in young (18–32 years) and older adults (63–78 years). We included cueing conditions to alert using a narrow or a wide attentional focus when comparing the composite objects, and used brief and relaxed exposure durations for stimulus presentation. Young adults showed large composite effects for faces, but none for watches. In contrast, older adults showed strong composite effects for faces and watches, albeit the effects were larger for faces. Moreover, composite effects for faces were larger for the wide attentional focus in both age groups, while the composite effects for watches of older adults were alike for both cueing conditions. Older adults showed low accuracy at the same levels for both types of stimuli when attended and non-attended halves were incongruent. Increasing presentation times improved performance strongly for congruent but not for incongruent composite objects. These findings suggest that the composite effects of older adults reflect substantial decline in the ability to control irrelevant stimuli, which takes effect both in non-face objects and in faces. In young adults, highly efficient attentional control mostly precludes interference of irrelevant features in novel objects, thus their composite effects reflect holistic integration specific for faces or objects of expertise.

## 1. Introduction

Many studies report age-related decline in tests of face recognition and face perception (Bartlett et al., 1989; Fulton and Bartlett, 1991; Crook and Larrabee, 1992; Searcy et al., 1999; Pfutze et al., 2002; Chaby et al., 2003; Hildebrandt et al., 2010; Germine et al., 2011). However, since there is also decline in other domains of cognitive functions which are necessarily involved in tests of face cognition, it is unclear whether the age-related decline concerns face-specific mechanisms, or rests on impairment in general spatial vision ability (Sekuler and Sekuler, 2000), processing speed (Salthouse, 1996), memory functions (Rajah and D'Esposito, 2005), or attentional control (Gazzaley et al., 2005a; Georgiou-Karistianis et al., 2006).

In several studies it was found that older adults suffer from deficits in tasks that require top-down suppression and attentional control (Gazzaley et al., 2005a,b, 2008). In perceptual tasks with simultaneous presentation of target and distracter stimuli it was found that, particularly, the ability to ignore irrelevant information suffers from aging (De Fockert et al., 2009; Quigley et al., 2010; Schmitz et al., 2010; Hanring et al., 2013; Geerligs et al., 2014). The loss of attentional control corresponds to the frontal lobe hypothesis of aging (West, 1996), since divided attention, attentional and executive control, and working memory function were found to be mediated by frontal brain areas (Goldman-Rakic, 1995; Cabeza et al., 1997; Fink et al., 1997; Rajah and D'Esposito, 2005; Prakash et al., 2009).

In recent psychophysical studies no age-related decline was reported for the composite face effect, which is a common index of holistic face processing (Konar et al., 2013; Meinhardt-Injac et al., 2014a). This led to the conclusion that holistic processing is preserved, or even a preferred vision mode at mature ages (ibid). Evidence for intact holistic processing of faces is at odds with findings obtained with tests on the ability to judge spatial-configural changes in faces, which is thought to be closely related to, or even an integral part of holistic face processing (Rossion, 2008). In several studies it was shown that older adults had difficulty to recognize two faces as different when the spatial distances of facial features were manipulated (Chaby et al., 2011; Meinhardt-Injac et al., 2015), which indicates a loss in spatial-configural processing of faces at mature ages (Daniel and Bentin, 2012). Using a face categorization task, Schwarzer et al. (2010) found that older adults did not prefer holistic to feature-based strategies. The overall picture of face processing in later adulthood is somewhat mixed, with studies supporting that face-specific abilities are maintained, while other studies report age-related decline in core capabilities of face processing (see also Hildebrandt et al., 2010, 2011).

The maintenance of the composite face effect at advanced ages deserves a second look, since the experimental measurement of the composite effect relies on the assumption that the observer has intact capabilities of attentional control, a domain that was shown to undergo strong age-related decline (see above). Generally, all experimental paradigms used to test whether objects are processed holistically or in a piecemeal manner share the common characteristic that holistic integration is concluded from the inability of the observer to judge a subset of object features (the attended or target features) independent of other object features (the unattended or context features, see Maurer et al., 2002 for an overview). Accordingly, holistic processing may be conceived as a failure to selectively attend objects parts (Richler et al., 2008; Richler and Gauthier, 2014). In later versions of the composite face paradigm holistic integration was concluded from the performance difference obtained for matching face halves in congruent and incongruent target to no-target relationships (congruency effect, CE), where only one half has to be attended and upper and lower half either agree (congruent) or disagree (incongruent) with respect to target face identity (Gauthier and Bukach, 2007; Cheung et al., 2008). Only if the observer is, in principle, able to selectively attend some object parts while ignoring others, the failure to do so with faces can be interpreted as indicating a specific processing mode exclusively elicited by faces, or objects of expertise after extensive training (Gauthier and Bukach, 2007). Measuring the composite effect for novel non-face objects has so far shown that there is only moderate or no interaction among attended and non-attended object parts when tested with healthy young adults (Farah et al., 1998; Gauthier et al., 2003; Richler et al., 2009a, 2011; Meinhardt et al., 2014).

At mature ages, however, the composite effect has so far not been tested with non-face control objects. Therefore, the finding of equal or even stronger face composite effects for older adults may not necessarily reflect intact holistic integration, but could reflect a general attentional age-related decline in the ability to suppress unattended object parts which provide conflicting target information. To clarify whether the composite effect for older adults is face-specific, or also exists for novel non-face objects is therefore mandatory. Testing face-specificity of the congruency effect by adding non-face control objects was a major aim of the present study.

**Figure 1:**
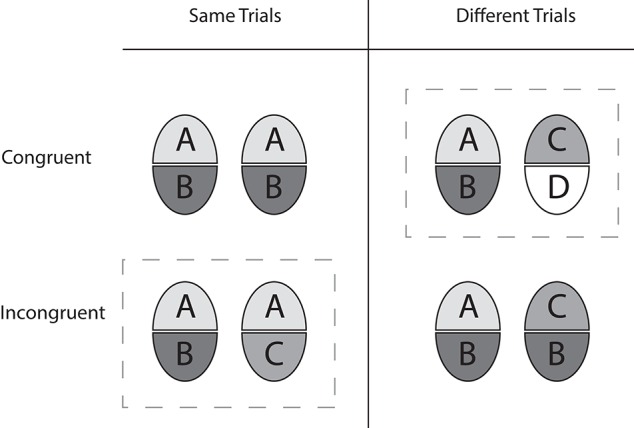
**Overview of the complete design, according to Cheung et al. (2008)**. The illustration shows the design for upper half matching. The dashed boxes mark the partial design as a subset of the complete design. In the partial design, “same” trials are always incongruent, agreeing in only the target halves, while “different” trials are always congruent, differing in both target and non-target halves. In the complete design, the number of same and different halve pairings is the same in congruent and in incongruent trials, and there is no confound of response alternative and congruency relation.

In the methodological debate about the proper measurement of the composite effect the design issue has become salient. As advocated by Richler and Gauthier (2014), it is important to use a fully balanced design with an equal frequency of same and different face half pairings (the “complete design,” CD) to avoid that observers show response bias, i.e., the preference of either the “same” or the “different” response category, due to formal characteristics of the design. If the CD is used, then the observation of response bias is informative, and can be attributed to characteristics of the observers, the stimulus material, and the experimental conditions. Recently, Meinhardt et al. (2014) suggested to use the CD, and to analyse response bias alongside accuracy in order to obtain a further clue toward the origin of the congruency effect. Because in trials with only part-based agreement of the face halves (incongruent trials) the “wholes” formed by integrating upper and lower halves are *always* different in the CD, while there is parity of same and different wholes in congruent trials (see Figure 1), the observer should more frequently respond “different” in incongruent trials, compared to congruent trials (“congruency bias,” CB), if she/he relies on representations of integrated whole objects rather than on independent representations of the two halves. This prediction from holistic processing characterizes the congruency effect qualitatively: If the composite objects are processed holistically, then *both* a congruency effect and a CB should be observed. This was indeed found for faces in young adults (Gao et al., 2011; Meinhardt et al., 2014). A CE alongside no congruency bias would indicate part based interference resulting from the inability to suppress the influence of the unattended halves, but no holistic integration. To characterize the interaction among halves for faces and non-face control objects with the CE and the CB for young and older adults was a second major aim of this study.

It has been outlined above that only if observers have intact attentional control the failure of selectively attending parts can be attributed to a holistic processing strategy. The congruency effect should depend on attentional constraints of a same/different discrimination task: If the observer knows from the beginning of the trial which halves, the upper or the lower, have to be compared, she/he can apply a narrow focus on the target parts, which should delimit the influence of the irrelevant halves. If, on the other hand, the observer does not know the target half at the first composite image presentation, and is informed later which halves are to be compared, she/he must encode the whole stimulus at study and try to narrow the focus at test. Hence, in the late cue condition, much more of the irrelevant halves is processed, which may potentially interfere with the judgment about the target halves. As expected, face congruency effects increase in the late cue condition, compared to the early cue condition (Meinhardt-Injac et al., 2014a). However, it is unclear whether interference among non-face object parts does in the same way depend on attentional focus conditions. Comparing the modulation of the CE by attentional focus conditions for faces and non-face objects can therefore give further valuable clues whether the interaction among object parts rests on the same or different mechanisms for both object categories. We therefore added the early cue / late cue manipulation to the experiment.

Further important constraints for the composite effect derive from temporal conditions and task difficulty. Studies on the composite effect that included variation of presentation times have shown that composite effects are present beginning with brief timings of about 50 ms in young adults (Richler et al., 2009b). However, this was not tested at older adults. Instead, most studies on holistic face perception used larger presentation times where settled performance could be expected (Boutet and Faubert, 2006; Konar et al., 2013). We used both brief and relaxed presentation times to compare the temporal constraints for holistic processing for young and older adults. Further, brief presentation times are a means to increase task difficulty remarkably for older adults (Salthouse, 1996). In recent studies it was revealed that older adults exhibit a strong overall “same” bias, which coincided with lower sensitivity (Meinhardt-Injac et al., 2014a, 2015). By varying presentation time we aimed at revealing how the composite effect and a potential response bias of older adults is linked to processing speed demands, and higher task difficulty.

In this study we systematically compared face and non-face matching performance, composite effects, and bias for young and older adults, using the outlined variation of attentional and temporal conditions. The results give important clues with respect to potentially different origins of the composite effect in young and older adults.

## 2. Materials and methods

### 2.1. Experimental outline

We used a same/different forced choice task, which required matching of a composite study stimulus, presented for 800 ms, and a composite test stimulus, presented afterwards for one of four possible presentation times (34, 84, 250, 650 ms). Subjects were informed by a cue which halves, the upper or the lower ones, had to be compared. They decided by button press whether study and test stimulus agreed or disagreed in the target halves (upper or lower). In the early cue condition, a target cue marking the half to be attended, was shown with the study image. In the late cue condition, the cue appeared after the study image, together with its subsequent mask. The two cue conditions were run in separate experimental blocks. Separate experiments were done with faces and watches, in random sequence chosen for each subject.

### 2.2. Design

We employed the “complete design” (CD) of the composite task (Cheung et al., 2008). In the CD congruent and incongruent stimulus half pairings are balanced, and, in contrast to the “partial” design, not confounded with response alternative (for details, see Richler and Gauthier, 2014). The CD and the partial design are illustrated in Figure 1. In incongruent trials, the non-target halves disagree when the target halves agree (“same”-trial), and agree when the target halves disagree (“different”-trial). In congruent trials both the target halves and the non-target halves either agree (“same”-trial), or disagree (“different”-trial). As a result of just part-based agreement in incongruent trials, the wholes formed by integrating upper and lower halves are always different. In congruent trials, there is parity of same and different whole objects (see Figure 1). The number of congruent and incongruent trials, as well as upper-half matching and lower half matching trials, was the same. The study comprised 2 stimulus categories (face/non-face) × 2 congruency relations (congruent/incongruent) × 2 cueing conditions (early/late) × 4 presentation times (34, 84, 250, 650 ms) = 32 conditions, which were administered to each young and older adult. Hence Stimulus, Congruency, Cue and Presentation Time were repeated measurement (within subjects) factors, while Age group was a between subjects factor.

### 2.3. Stimuli

#### 2.3.1. Face stimuli

**Figure 2:**
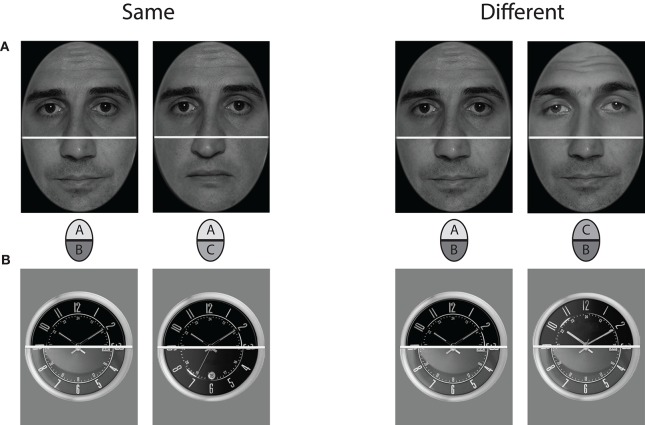
**Stimulus examples for upper stimulus half comparison in incongruent trials (lower row of Figure 1), for faces (A) and watches (B)**. The left composite stimulus pairs show same upper halves combined with different lower halves, the right ones show different upper halves combined with same lower halves. Note that the integrated wholes of both halves are different in both “same” and “different” trials.

Face half stimuli were constructed from 20 pictures of male german and swiss models, taken in a photo studio under controlled lighting conditions. Photos were frontal view shots of the whole face. The original images were edited with Adobe Photoshop CS4 software to create face half sets. Photographs were initially converted to 8 bit grayscale pictures and superimposed with an elliptical frame mask to obliterate all external facial features, such as hair, ears, or chin line. The elliptical cutouts were then split horizontally at the bridge of the nose, thus yielding 20 upper and 20 lower face halves. Each upper half was recombined with three lower halves to constitute a final set of 60 compound faces. The cutline between the face halves was hidden with a superimposed white bar 5 pixels in thickness. It was warranted that any upper face part was never recombined with the lower half of the same original face, thus there was no replication of the same full face in the experimental trials. Additionally, each of the 20 lower and upper halves appeared exactly three times in the final set of stimuli. Stimulus examples are shown in Figure 2.

#### 2.3.2. Non-face stimuli

Twenty watches were sampled from internet sources, and selected such that they had high overall resemblance, showed the same time, and had non-salient distinctive single features within the clock-face. The images were transformed to gray and matched on lightness and contrast. The cutline for subdividing into upper and lower halves was exactly through the midpoint of the clock-face. All external features were removed, and a circular frame which was identical for all stimuli was superimposed on the clock-face. Stimulus examples are shown in Figure 2. As for the faces, a final set of 60 composite watches was constructed.

### 2.4. Subjects

Overall, 32 young adults and 28 older adults participated. All participants had normal or corrected to normal vision and reported normal neurological and psychiatric status. Young adult subjects were undergraduate students. The mean age of the student group was 22.8 years (range 18–32 years), and 69% were female. Participants received course credit points for participation, or payment. The mean age in the older adults sample was 69.4 years, age range was 63–78 years, and 53% of the participants were female. The older adults subjects were recruited from a database of members from the “Studieren 50+” programme of the University of Mainz. Accordingly, all had at least highschool level education. Two subjects were still in profession, the others retired. All older adults lived in the area of Mainz, and lived independent lives. They were paid for participation. The mini-mental state examination (MMSE; Folstein et al., 1975) was used to evaluate the mental status. All subjects passed the test with more than 27 of the 30 points.

### 2.5. Apparatus

The experiment was executed with Inquisit runtime units. Stimuli were displayed on NEC Spectra View 2040 TFT displays in 1280 × 1024 resolution at a refresh rate of 60 Hz. Screen mean luminance *L*_0_ was 100 cd/m^2^ at a michelson contrast of (*L*_*max*_ − *L*_*min*_)/(*L*_*max*_ + *L*_*min*_) = 0.98 and practically dark background (about 1.4 cd/m^2^). No gamma correction was used. The room was darkened so that the ambient illumination approximately matched the illumination on the screen. Stimulus size was 250 × 350 pixels (width × height). The stimuli were viewed binocularly at a distance of 70 cm. Subjects used a distance marker but no chin rest throughout the experiment. The subjects responded via an external key-pad (Cedrus RB-830 response pad).

### 2.6. Preparation and preliminary measurements

Preliminary measurements were taken and former results for young and older adults (see Meinhardt et al., 2014; Meinhardt-Injac et al., 2014a) were used to guide parameter settings. The difficulty of matching watch stimuli was manipulated by exchanging stimulus objects until a matching accuracy in congruent trials with early cue of 90% correct was achieved by the young adults at stimulus durations of 250 ms. This matched the performance obtained with face stimuli fairly good. Previous results showed that older adults met the 90% correct level with faces for presentation times of beyond 600 ms (Meinhardt-Injac et al., 2014a). Because differential performance with both stimulus classes is a potential age-related effect we did not adjust difficulty of watch matching by manipulating stimuli for the older adults group. The longest presentation time was set to 650 ms, since no further improvement was observed in previous testing for older adults even for longer durations. Adding the two brief timings of 34 and 84 ms to 250 and 650 ms we expected to get a good sampling both of the rising and the saturating part of the sensitivity vs. presentation time function, since this function is known to show strong rise for the first 100 ms and then starts to saturate gradually (see Richler et al., 2009b).

### 2.7. Procedure

Subjects were instructed that just the cued halves had to be compared, but that the uncued halves could also agree od disagree. They were also instructed to judge as accurately as possible, and that there was no speed pressure for the response. The temporal order of events in a trial was: fixation mark (750 ms), blank (300 ms), study stimulus (800 ms), mask (400 ms), blank (800 ms), test stimulus (34, 84, 250, or 650 ms), mask (400 ms), and blank frame until response. In the early cue condition a rectangular bracket marking the target stimulus half was shown together with the study stimulus, and remained until the test stimulus was masked. In the late cue condition the cue presentation began with the mask of the study stimulus. Stimulus position jittered randomly within a region of ±50 pixels around the center of the screen to preclude image region matching strategies between two subsequent stimulus presentations. Masks were constructed from scrambled 5 × 5 pixel blocks of the stimulus shown before. No feedback about correctness was given.

Young adults were made familiar with the task by responding to some randomly selected probe trials. Older adults were carefully prepared for the experiment. First, paper print examples of the stimulus pairings were explained to the subject. The experimenter displayed paper prints of 10 stimulus pairs, and asked participants to name the five pairs showing objects with the same upper halves and the five showing different upper halves. Subjects were given as much time as needed to label the 10 pairs. If errors occurred, the experimenter adverted to the wrongly labeled pairs and drew attention to just the halves to be compared. The first minutes at the computer were spent on just congruent trials presented with the longest presentation time (650 ms), which all subjects could do with good accuracy. The subjects then responded to probe trials of the experiment with congruent and incongruent trials for about 8 min. After the preparation phase the experimental blocks started.

Two experiments were run, one with faces, and one with watches. One experiment comprised 16 conditions (see Design). Each condition was measured with 16 same—and 16 different—trials. Eight of these *N* = 16 replications were done with upper half, and 8 with lower half as the target. The total 512 trials were subdivided into two blocks, 256 early cue and 256 late cue trials. Going through a block took about 20 min. Interleaved by a brief pause, the two blocks were administered on a single day, one with early cue, and one with late cue, in random order across subjects. The two experiments were done at two consecutive days.

### 2.8. Performance measures and data analysis

Performance was assessed within the framework of the signal detection paradigm. Based on the relative frequencies for the two response categories in same and different trials, the sensitivity measure

(1) d′=z(Hit)−z(FA)

and the estimate of the response criterion

(2) $$c=-\frac{z(Hit)+z(FA)}{2}$$

was calculated (see MacMillan and Creelman, 2005, p. 8 and p. 29). The hit-rate (Hit) was defined as the rate of correctly identifying same target halves and the correct rejection rate (CR) was defined as the rate of correctly identifying different target halves. False alarm rate (FA) and the rate of misses (Miss) were defined as the complementary rates to CR and Hit, respectively. Perfect or zero hit or false alarm rates were corrected before transforming to *d*′, replacing the rate by *p* = 1 − 1/(2*N*), or *p* = 1/(2*N*), respectively (see MacMillan and Creelman, 2005, p. 8). For further analyses of the sensitivity measure congruency effects were calculated as the difference measure *CE* = *d*′(CC) − *d*′(IC). Here, congruent is abbreviated as CC (congruent composite), and incongruent as IC (incongruent composite). Congruency effects were also calculated for the response criterion according to *CB* = *c*(*IC*) − *c*(*CC*) to measure the effect of congruency on response bias (see Section 1). Note that, with the given convention for defining the four events of the forced choice task, positive values of *c* indicate a “different” bias and negative values a “same” bias.

Further, we provide a bias measure in terms of the error proportion of wrong “different” responses:

(3) $$q=\frac{Miss}{Miss+FA}.$$

If *q* = 0.5, then both responses occur with equal likelihood. A ratio of *q* > 0.5 indicates a tendency to respond “different” while *q* < 0.5 indicates a preference toward “same” responses. To compare response preferences for congruent and incongruent trials we also calculated odds ratios for both types of errors, i.e.,

(4) $$OR=\frac{Miss/Hit}{FA/CR}.$$

The odds ratio (Equation 4) indicates how much larger the odds are for wrong “different” responses compared to wrong “same” responses.

Both the *d*′ and the *c* measure were analyzed with ANOVA.

## 3. Results

### 3.1. Sensitivity measure

**Figure 3:**
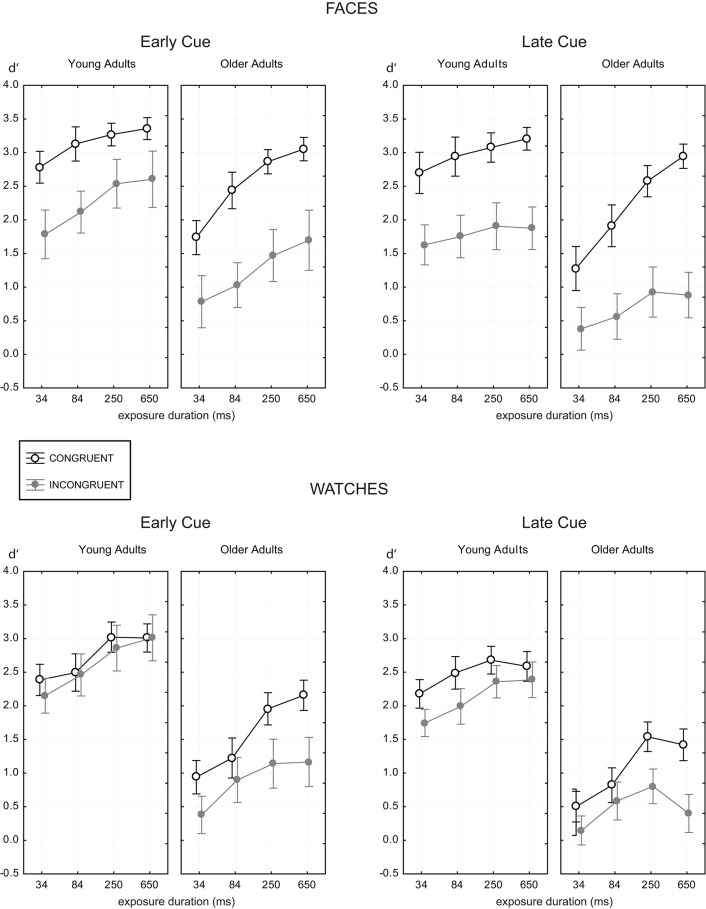
**The *d*′ measure as a function of presentation time for the two age groups with faces (upper panels) and watches (lower panels), and target half cue given at study image (early cue, left panels) and before test image (late cue, right panels)**. Data for the congruent trials are shown as open black circles, gray symbols indicate data for incongruent trials. Error bars indicate 95% confidence limits of the means.

Figure 3 shows *d*′ means as a function of presentation time for all experimental conditions. Generally, there were striking age-related differences in performance level, and its dependency on presentation time and stimulus category. Data analysis using ANOVA revealed significance of all main effects, i.e., age [$F_{\left(1,58\right)}=155.4,\;p<0.001,\eta_{p}^{2}=0.73$], stimulus [$F_{\left(1,58\right)}=37.0,p<0.001,\eta_{p}^{2}=0.39$], cue [$F_{\left(1,58\right)}=110.5,p<0.001,\eta_{p}^{2}=0.66$], congruency [$F_{\left(1,58\right)}=217.1,p<0.001,\;\eta_{p}^{2}=0.79$], and presentation time [$F_{\left(3,174\right)}=185.2,p<0.001,\eta_{p}^{2}=0.76$]. These effects were analysed in detail by considering first and higher order interactions. Because sensitivity was mostly settled for presentation times of 250 ms and beyond in both age groups, we provide tables with pairwise comparisons for data agglomerated over the last two presentation times. In these tables we report age-related performance differences, as well as congruency effects for stabilized performance levels.

#### 3.1.1. Stimulus effects

There was an age × stimulus interaction [$F_{\left(1,58\right)}=24.2,p<0.001,\eta_{p}^{2}=0.29$], which indicated different performance with both stimulus categories in either age group. Comparing sensitivity across stimuli for young adults showed that performance was at equal levels with both stimulus categories [*F*_(1, 58)_ = 0.72, *p* = 0.398], while older adults performed notably worse with watches compared to faces [*F*_(1, 58)_ = 56.75, *p* < 0.001]. These effects did not depend on presentation time [presentation time × stimulus interaction *F*_(3, 174)_ = 1.10, *p* = 0.351], and were also present in the data for the two longest timings [young adults: *F*_(1, 58)_ = 0.01, *p* = 0.937; older adults: *F*_(1, 58)_ = 56.47, *p* < 0.001]. More detailed analysis revealed how stimulus effects differed in congruent and incongruent trials. For young adults there was better performance with watches in incongruent trials [$\Delta d^{\prime }=-0.33,t_{\left(31\right)}=-2.38,p<0.03$], but better performance with faces in congruent trials [$\Delta d^{\prime }=0.47,t_{\left(31\right)}=7.44,p<0.001$]. Hence, agglomerated across congruency, both effects canceled out. For older adults, in contrast, performance was much better with faces than with watches in congruent trials [$\Delta d^{\prime }=1.05,t_{\left(27\right)}=7.44,p<0.001$], but not significantly different in incongruent trials [$\Delta d^{\prime }=0.30,t_{\left(27\right)}=2.00,p=0.055$]. Again, these results did not change when only the last two timings were considered [young adults, congruent: $\Delta d^{\prime }=0.42,t_{\left(31\right)}=9.39,p<0.001$; young adults, incongruent: $\Delta d^{\prime }=-0.41,t_{\left(31\right)}=-2.28,p<0.03$; older adults, congruent: $\Delta d^{\prime }=1.12,t_{\left(27\right)}=23.18,p<0.001$; older adults, incongruent: $\Delta d^{\prime }=0.39,t_{\left(27\right)}=2.02,p=0.053$].

#### 3.1.2. Age-related performance differences

Young adults showed higher matching accuracy in all conditions of the experiment. The age × stimulus interaction [$F_{\left(1,58\right)}=24.2,p<0.001,\eta_{p}^{2}=0.29$] reflected that age-related performance differences were much stronger with watches than with faces. The strong age × stimulus interaction was maintained when only the last two presentation times were considered [$F_{\left(1,58\right)}=16.0,p<0.001,\eta_{p}^{2}=0.22$]. Table 1 lists the results of pairwise tests for these data. For faces, age-related performance differences reached an average of 0.68 *d*′ units, with an effect size of *d* = 0.98. For watches, a difference of 1.43 *d*′ units was obtained, with an effect size of *d* = 2.32. Table 1 also shows that age-related sensitivity differences, measured in *d*′ units, were much larger (at least doubled) in incongruent compared to congruent trials. However, due to the much larger standard errors in incongruent trials, this effect did not become obvious in the effect size measure *d*.

**Table 1:** **Age-related performance differences agglomerated across the two longer presentation times**.

**Stimulus**	**Cue**	**Congruency**	**Δ*d*′**	***s_e_***	***t***	***df***	***p***	***d***
Faces	Early	CC	0.36	0.09	4.00	58	0.001	1.04
Faces	Early	IC	0.99	0.27	3.68	58	0.001	0.95
Faces	Late	CC	0.38	0.12	3.07	58	0.003	0.79
Faces	Late	IC	0.99	0.23	4.39	58	0.001	1.14
Mean			0.68					0.98
Watches	Early	CC	0.97	0.14	6.95	58	0.001	1.80
Watches	Early	IC	1.79	0.23	7.66	58	0.001	1.98
Watches	Late	CC	1.16	0.11	10.11	58	0.001	2.62
Watches	Late	IC	1.78	0.16	11.08	58	0.001	2.87
Mean			1.43					2.32

*The table shows d′ difference, its standard error, t-value, degrees of freedom, significance level, and Cohen's d*.

There were further significant interactions of factors with age, which involved congruency and presentation time (see below).

#### 3.1.3. Congruency effects

There were large congruency effects, which were notably larger for faces than for watches [congruency × stimulus, $F_{\left(1,58\right)}=62.62,p<0.001,\eta_{p}^{2}=0.52$]. Congruency effects were also modulated by age, having larger CEs for older than for younger adults [congruency × age, $F_{\left(1,58\right)}=11.24,p<0.002,\eta_{p}^{2}=0.16$]. The congruency effect also depended on cueing, with larger CEs for the late compared to the early cue [congruency × cue, $F_{\left(1,58\right)}=6.71,p<0.02,\eta_{p}^{2}=0.1$]. The congruency effect also depended on presentation time, but in different ways for the two age groups (see below).

When analysing the data of only the last two presentation times, all the reported interactions were maintained [congruency × stimulus, $F_{\left(1,58\right)}=19.07,p<0.001,\eta_{p}^{2}=0.25$; congruency × age, $F_{\left(1,58\right)}=4.30,p<0.05,\eta_{p}^{2}=0.07$; congruency × cue, $F_{\left(1,58\right)}=8.95,p<0.01,\eta_{p}^{2}=0.13$], but two further higher order interactions emerged. For settled performance levels, there was a significant congruency × cue × stimulus interaction [$F_{\left(1,58\right)}=7.68,p<0.01,\eta_{p}^{2}=0.11$], and a significant congruency × stimulus × age interaction [$F_{\left(1,58\right)}=7.68,p<0.01,\eta_{p}^{2}=0.11$].

Table 2 lists the results of testing congruency effects for the last two presentation times, which illuminates these interactions. With faces, both young and older adults showed large congruency effects of about one *d*′ unit (young adults), and beyond 1.3 *d*′ units (older adults). With watches young adults showed no substantial CEs. There was a modest congruency effect only in the late cue condition (0.26 *d*′ units), but no congruency effect in the early cue condition. Older adults, in contrast, showed strong congruency effects for watches of nearly one *d*′ unit in both cue conditions. The CEs for watches had large effect sizes of more than one Cohen's *d*, and compared to the CE found for young adults with faces in the early cue condition. Pairwise comparisons across age showed that older adults had significantly larger CEs than young adults for both faces and watches. This was consistently found for early and late cueing (see last column of Table 2). The significant congruency × cue × stimulus was reflected by larger CEs for the late compared to the early cue, which applied to faces, but not to watches.

**Table 2:** **Congruency effects (CEs), for both age groups and stimulus classes, agglomerated across the two longer presentation times**.

**Age group**	**Stimulus**	**Cue**	***CE***	***s_e_***	***t***	***df***	***p***	***d***	**Effect size**	**Older-younger**
Young adults	Faces	Early	0.75	0.18	4.19	31	0.001	0.74	Large	^*^
Young adults	Faces	Late	1.26	0.17	7.28	31	0.001	1.29	Large	^*^
Young adults	Watches	Early	0.08	0.15	0.52	31	0.605	0.09	–	^***^
Young adults	Watches	Late	0.26	0.12	2.19	31	0.036	0.39	Medium	^***^
Older adults	Faces	Early	1.38	0.19	7.27	27	0.001	1.37	Large	
Older adults	Faces	Late	1.87	0.18	10.10	27	0.001	1.91	Large	
Older adults	Watches	Early	0.91	0.16	5.51	27	0.001	1.04	Large	
Older adults	Watches	Late	0.89	0.13	6.90	27	0.001	1.30	Large	

The table shows the CE, its standard error, t-value, degrees of freedom, significance level, and Cohen's d with classification of effect size. The last column indicates the significance level for comparing CEs in the same conditions across age (

* *α = 0.05*,

*** *α = 0.001)*.

#### 3.1.4. Effects of early or late target cue

The cueing manipulation modulated performance strongly, yielding better performance for the early compared to the late target cue. These effects were similar for both age groups [cue × age, *F*_(1, 58)_ = 2.08, *p* = 0.155] and both stimulus classes [cue × stimulus, *F*_(1, 58)_ = 0.27, *p* = 0.606]. Early vs. late cueing affected the CE (s.a.), and its effects depended on presentation time (see below).

#### 3.1.5. Effects of presentation time

The strong effect of presentation time (s.a.) was different in the two age groups [presentation time × age, $F_{\left(3,174\right)}=10.51,p<0.001,\eta_{p}^{2}=0.15$]. Older adults showed more improvement with increasing presentation time, while young adults were closer to their settled performance even at brief timings. The effect of cueing was also modulated by presentation time, with larger performance differences for early and late cue occurring at the two longer presentation times [presentation time × cue, $F_{\left(3,174\right)}=4.60,p<0.01,\eta_{p}^{2}=0.07$].

The congruency effect also depended on presentation time [presentation time × congruency, $F_{\left(3,174\right)}=5.62,p<0.001,\eta_{p}^{2}=0.09$]. This effect was moderated by age. While the congruency effect was constant across presentation time for young adults, it increased with increasing presentation time for older adults [presentation time × congruency × age, $F_{\left(3,174\right)}=12.10,p<0.001,\eta_{p}^{2}=0.17$]. Among all the differential effects involving presentation time, this effect had largest effect size. This age-differential effect was due to the fact that older adults showed more improvement with increasing presentation time in congruent trials, but could not improve at the same rate in incongruent trials (see Figure 3). Young adults, instead, showed improvement at similar rates in both congruency conditions, with a marginal tendency toward stronger improvement in incongruent trials.

### 3.2. Response bias

**Figure 4:**
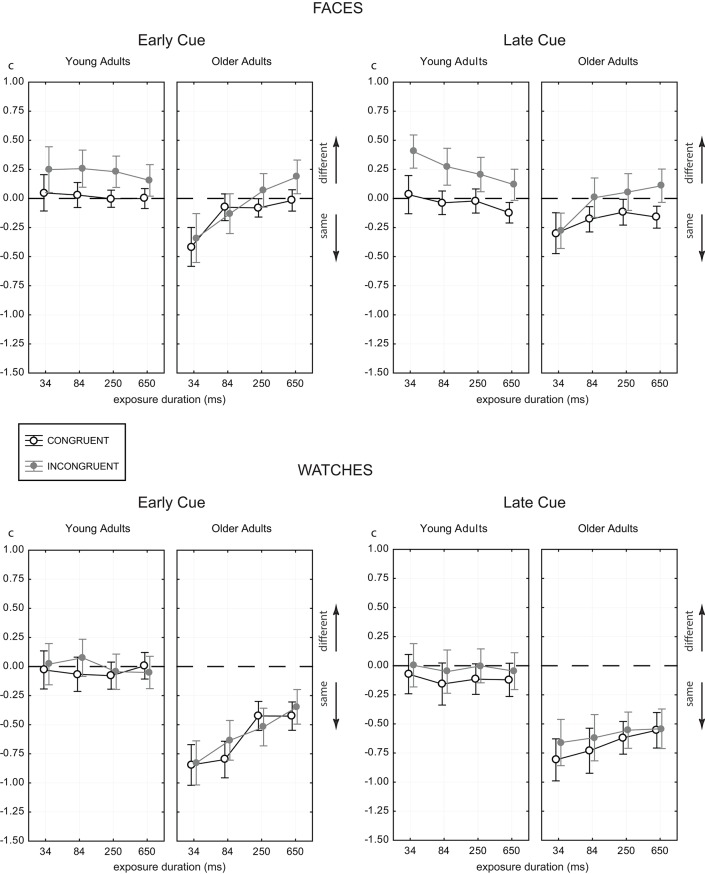
**Estimated response criterion *c* as a function of presentation time for the two age groups for faces (upper panels) and watches (lower panels), and target half cue given at study image (early cue, left panels) and before test image (late cue, right panels)**. Conventions as in Figure 3.

Figure 4 shows the mean estimates of the response criterion *c* as a function of presentation time for all experimental conditions. ANOVA revealed main effects of presentation time [$F_{\left(3,174\right)}=9.64,p<0.001,\eta_{p}^{2}=0.14$], congruency [$F_{\left(1,58\right)}=73.51,p<0.001,\eta_{p}^{2}=0.559$], stimulus [$F_{\left(1,58\right)}=56.80,p<0.001,\eta_{p}^{2}=0.49$], and age [$F_{\left(1,58\right)}=50.46,p<0.001,\eta_{p}^{2}=0.465$], but no effect of cueing [*F*_(1, 58)_ = 2.66, *p* = 0.11]. The main effect of age indicated that older adults had consistently lower values in the response criterion than young adults in all experimental conditions (see Figure 4). However, there was a strong age × stimulus interaction [$F_{\left(1,58\right)}=15.99,p<0.001,\eta_{p}^{2}=0.22$], which indicated that the response criterion used by young adults for watches was only marginally smaller than for faces, while older adults strongly preferred “same” responses for watches, but not for faces (see Figure 4).

A further striking difference of young and older adults was the strong differential effect of presentation time [presentation time × age, $F_{\left(3,234\right)}=26.21,p<0.001,\eta_{p}^{2}=0.31$]. For older adults the response criterion increased with presentation time, i.e., the strong “same” bias continuously diminished with increasing presentation time. For young adults, in contrast, the response criterion slightly decreased with increasing presentation time, or stayed relatively constant about zero.

Figure 4 also shows that the response criterion *c* reached settled values for the two longer presentation times. For these timings we compared the response criterion across age for both stimuli, cues and congruency relations (see Table 3). The pairwise tests reveal that there were no significant age-related differences in the response criterion for faces. For watches, there were strong age-related differences, which were quite constant across congruency and cueing. These effects were due to the strong “same” bias of older adults for watches, which did not vanish even at longer presentation times.

**Table 3:** **Age-related differences in the response criterion, *c*, agglomerated across the two longer presentation times**.

**Stimulus**	**Cue**	**Congruency**	***Δc***	***s_e_***	***t***	***df***	***p***	***d***
Faces	Early	CC	0.05	0.05	1.03	58	0.307	0.27
Faces	Early	IC	0.07	0.09	0.74	58	0.462	0.19
Faces	Late	CC	0.07	0.06	1.12	58	0.265	0.29
Faces	Late	IC	0.08	0.09	0.92	58	0.361	0.24
Mean			0.07					0.25
Watches	Early	CC	0.39	0.08	5.11	58	0.001	1.32
Watches	Early	IC	0.39	0.10	4.06	58	0.001	1.05
Watches	Late	CC	0.47	0.09	5.25	58	0.001	1.36
Watches	Late	IC	0.53	0.10	5.28	58	0.001	1.37
Mean			0.44					1.27

*The table shows c difference, its standard error, t-value, degrees of freedom, significance level, and Cohen's d*.

#### 3.2.1. Congruency bias (CB)

The strong modulation of the response criterion by the congruency relation indicated larger values of *c* in incongruent trials, compared to congruent trials, i.e., a CB effect. The CB was moderated by age [congruency × age, $F_{\left(1,58\right)}=4.25,p<0.05,\eta_{p}^{2}=0.07$] and, notably, by stimulus [congruency × stimulus, $F_{\left(1,58\right)}=19.45,p<0.001,\eta_{p}^{2}=0.25$]. The congruency × age interaction indicated that the CB was larger for younger than for older adults. However, this was due to the fact that younger adults showed a CB already at brief timings, while, for older adults, the CB emerged at larger presentation times.

Analysing the response criterion data at only the last two presentation times showed that the congruency × age interaction vanished [$F_{\left(1,58\right)}=0.30,p=0.587,\eta_{p}^{2}=0.01$], indicating equal CB effects for relaxed timings. Table 4 shows the CB agglomerated for the last two presentation times, and in detail for both stimuli, cues and the two age groups. For watches, there was no CB in either age group, and for both early and late cueing. For faces, there were significant CB effects, which reflected a similar congruency modulated criterion shift in both age groups, and for both the early and the late cue. The CB reached effect sizes in a span of *d* = [0.63, 0.82] which indicated similar CB effects for both age groups and with both cues for faces.

**Table 4:** **Congruency bias effects (CBs), for both age groups and stimulus classes, agglomerated across the two longer presentation times**.

**Age group**	**Stimulus**	**Cue**	***CB***	***s_e_***	***t***	***df***	***p***	***d***	**Effect size**	**Older-younger**
Young adults	Faces	Early	0.20	0.05	3.92	31	0.001	0.69	Large	n.s.
Young adults	Faces	Late	0.24	0.05	4.65	31	0.001	0.82	Large	n.s
Young adults	Watches	Early	−0.01	0.04	−0.28	31	0.784	0.05	–	n.s.
Young adults	Watches	Late	0.09	0.05	1.88	31	0.069	0.33	–	n.s.
Older adults	Faces	Early	0.18	0.05	3.33	27	0.003	0.63	Medium	
Older adults	Faces	Late	0.22	0.05	4.12	27	0.001	0.78	Large	
Older adults	Watches	Early	−0.01	0.05	−0.17	27	0.869	0.03	–	
Older adults	Watches	Late	0.04	0.05	0.74	27	0.468	0.14	–	

*The table shows the CB, its standard error, t-value, degrees of freedom, significance level, and Cohen's d with classification of effect size. The last column indicates the significance level for comparing CEs in the same conditions across age, n.s., not significant*.

To illuminate the different kinds of errors made at the last two presentation times we calculated the error proportion *q*, and report the odds ratio for wrong “different” compared to wrong “same” responses. Table 5 shows the results. The data reveal that, albeit older adults made much more errors of both kinds in incongruent trials than young adults, the error proportion *q* for faces was modulated by the congruency relation similarly for young and older adults. The odds ratios indicate that the risk for wrong “different” responses to faces was about doubled in incongruent compared to congruent trials in both age groups. For watches, there was only a marginally higher risk for wrong “different” responses in incongruent trials for both young and for older adults. This illustrates the quite similar effect of congruency on response bias in both age groups, which was found albeit the overall response bias for watches differed strongly among both age groups (see *q* measure in Table 5). Further, the proportion correct rates shown in Table 5 illustrate that older adults reached good performance with faces in congruent contexts (92% correct judgments), coming close to the performance of young adults (95% correct judgments).

**Table 5:** **Bias measure *c*, error rates and error proportion *q*, for both age groups and stimulus classes, agglomerated across the two longer presentation times**.

**Age group**	**Stimulus**	**Congruency**	***c***	***CR***	***FA***	***Hit***	***Miss***	***p_c_***	***q***	***OR***	***q_OR_***
Young adults	Faces	CC	−0.04	0.94	0.06	0.95	0.05	0.95	0.46	0.85	2.29
Young adults	Faces	IC	0.18	0.90	0.10	0.83	0.17	0.87	0.64	1.94	
Young adults	Watches	CC	−0.08	0.91	0.09	0.93	0.07	0.92	0.43	0.73	1.19
Young adults	Watches	IC	−0.04	0.90	0.10	0.91	0.09	0.91	0.47	0.87	
Older adults	Faces	CC	−0.10	0.91	0.09	0.94	0.06	0.92	0.41	0.68	2.10
Older adults	Faces	IC	0.10	0.77	0.23	0.70	0.30	0.73	0.56	1.42	
Older adults	Watches	CC	−0.51	0.64	0.36	0.92	0.08	0.78	0.19	0.16	1.20
Older adults	Watches	IC	−0.49	0.48	0.52	0.82	0.18	0.65	0.25	0.20	

*The table shows c, the rates for CR, FA, Hit, and Miss, proportion correct, p_c_, the error proportion measure, q, the odds ratio for Miss compared to FA, OR, and the ratio of the OR for incongruent, compared to congruent trials, q_OR_*.

## 4. Discussion

We studied face and non-face object perception with the complete design of the composite paradigm to reveal face-specificity of congruency effects in young and older adults. We found that congruency effects were face-specific in young, but not in older adults. Congruency effects of older adults increased with increasing presentation time, and were substantial also for novel non-face objects for relaxed exposure durations where performance reached settled levels. In the following we discuss these results with respect to the potentially different origins of the congruency effect in young and older adults, and in the context of other recent findings.

### 4.1. The effect of presentation time on the congruency effect for young and older adults

An important characteristic of the CEs of older adults is their dependency on presentation time. The CEs of older adults increased with increasing exposure durations, while the CEs of young adults were strong even at the shortest presentation times, and tended to decline afterwards. The CEs of older adults at longer presentation times are the result of the differential improvement in congruent and incongruent trials. With incongruent composites there was hardly improvement, while performance improved at strong rates for congruent composites, and stronger for faces than for watches. This means that older adults could benefit from larger temporal processing resources only in the condition where attentional control of the irrelevant halves and focusing only the target parts was not required. There, face processing showed stronger benefit than watch processing, finally reaching good levels close to the levels of young adults.

Stimulus processing in trials requiring to control irrelevant information stayed at same modest levels for faces and watches. This gives important clues to the origin of the congruency effect. Apparently, older adults had difficulty to control the effects of the irrelevant halves, independent of object category. Younger adults had no particular problems in this respect. Performance increased with presentation time in incongruent trials with at least the rate found in congruent trials, and reached levels that were largely different from the levels reached by older adults (see Figure 3 and Table 1). These results confirm earlier results showing that the congruency effects are already present at brief timings for young adults (Richler et al., 2009b). Further, young adults were able to use additional processing resources to delimit the influence of irrelevant features (Meinhardt-Injac et al., 2011). In this study, young adults could control incongruent watch halves fairly well at larger presentation times, and even in the late cue condition, thus congruency effects were true face-specific effects in this age group. Good control of irrelevant information is further indicated by the fact that young adults reached better performance in incongruent trials with watches than with faces (see Section 3.2). This indicates the different origins of performance with incongruent composite faces and watches in young adults. Incongruent features could be controlled fairly well with watches, while irrelevant face halves could not be ignored. This result clearly suggests a specific, integrative mode of processing exclusively for faces, but not watches.

In contrast to young adults, the stimulus unspecific performance loss of older adults with incongruent composites points to a general impairment in controlling irrelevant features. This results adds to the age differential results found in other studies where interference of non-attended scenes on attended faces, and vice versa, was measured (Gazzaley et al., 2008; Quigley et al., 2010; Schmitz et al., 2010). Moreover, age-related decline in controlling irrelevant features as a determinant of worse performance in incongruent trials is supported by the results obtained for the bias measure.

### 4.2. The role of response bias

Analysis of response bias revealed that the congruency effects for faces of both age groups were accompanied by more wrong “different” responses in incongruent, compared to congruent trials (CB effect). For young adults, this was consistently observed for all presentation times, while, for older adults, the CB emerged only for settled performance at relaxed presentation times. The CB shows that the observers were more strongly biased to respond “different,” in agreement with the prediction from holistic processing (see Introduction). Hence, for relaxed timings, we found both a CE and a CB for young and for older adults, which is agreement with holistic processing of faces in both age groups.

The CE for watches of the older adults, however, was not accompanied by a CB. Detailed analysis of the kind of errors showed that wrong “same” responses were more likely than wrong “different” responses with watches, and this was not modulated by the congruency relation. Hence, the fact that all whole watches were different in incongruent trials did not influence the response behavior of older adults. This means that the errors made in incongruent trials with watches root in part based interference rather than in holistic integration of upper and lower halves.

For older adults we found significant CEs for watches, but also *larger* CEs for faces, compared to young adults (see Table 2). The differential pattern of CBs for faces and watches, gives a clue to interpreting this result. Note that the CE increases when more errors are made in incongruent, compared to congruent trials, irrespective of the kind of errors. Albeit young and older adults have a similar CB for faces, older adults made much more errors of *both* kinds, i.e., also the frequency of wrong “same” responses increased in incongruent trials. That is, also for faces there were more errors which were not induced by the non-identity of the wholes, but by part based interference. Hence, the stronger CEs of older adults for faces does not indicate stronger reliance on holistic processing strategies, but a plus in interference of parts. This supports the conclusion that the larger CE of older adults for faces and the CE for watches have a common ground in larger part based interference from the non-attended parts in composite objects. The stronger susceptibility to part-based interference indicates a loss in efficient attentional control that applies to both object categories.

A further striking observation was the strong general “same” bias of older adults, a much stronger one for watches than for faces, which diminished with increasing presentation times. Both the stimulus dependency and the dependency on presentation time indicated that the “same” bias of older adults was performance related. That is, older adults preferred to respond “same” when they experience high degrees of uncertainty about the correct judgment, i.e., experienced task difficulty is high. This is in agreement with earlier findings of a tendency to overlook diagnostic differences (Daniel and Bentin, 2012; Meinhardt-Injac et al., 2014b, 2015), and corresponds to the typical failure of older adults to categorize new objects as known ones (Fulton and Bartlett, 1991; Lee et al., 2014).

### 4.3. Age-related decline in face-specific processing

The findings of the present study bring up the question whether the observation of comparable face composite effects for young and older adults justifies the conclusion that the specific mechanisms of holistic face processing are intact, and do not undergo age-related decline, as claimed recently (Konar et al., 2013; Meinhardt-Injac et al., 2014a). As the face-unspecific CEs in older adults, as well as the differential CB effects show, comparable face composite effects for young and older adults are no solid grounds for this conclusion. A significant proportion of the face congruency effects observed for older adults may root in face-unspecific and part-based interference, but not in integrative processing specific for faces. While CEs associated with CB effects were observed in older and young adults, which indicates holistic integration for faces in both age groups, the observation of face-unspecific CEs for older poses severe constraints on the interpretation of the face CEs in older adults, since it can hardly be determined to which extent these effects reflect part interference on the one and holistic integration on the other hand.

We therefore conclude that strong composite effects for faces are no sufficient evidence to concluding intact face-specific processing at advanced ages. In this study, older adults performed much better with faces than with watches, but only for congruent composites where attending target parts and attending non-target parts yields same results. This supports that older adults used a global viewing strategy, which is advantageous for faces, but not for non-face objects, which differ in single features, but hardly in global appearance. We think the relatively good performance reached with congruent face composites is no sufficent proof for intact and efficient holistic processing of faces. A major advantage of holistic processing is that changes in inner face details have strong effects on the overall facial appearance. However, there is evidence that older adults rely on global face shape (Schwarzer et al., 2010) and have difficulty judging the inner face details (Meinhardt-Injac et al., 2014b). Holistic integration of facial cues from different facial areas is also important in emotion recognition. Using the bubbles technique Smith et al. (2005) revealed that happiness and anger may be readily recognized just from single face regions (happiness from the mouth and anger from the eyes region), but to categorize the remaining four emotions correctly, cues from more than one area have to be integrated. However, aging studies of emotion recognition consistently report age-related deficits in identifying particularly anger, fear and sadness (Sullivan and Ruffman, 2004), and also declined capabilities in inferring emotions from the eyes region and the whole face (Sullivan et al., 2007). Further, the reported strong age-related decline in using spatial-configural cues (Chaby et al., 2011; Meinhardt-Injac et al., 2015), and findings of reduced grouping ability for face fragments into whole intact faces (Norton et al., 2009) point toward impairment in core-capabilities of holistic processing.

### 4.4. Conclusion

Using the complete design of the composite paradigm with faces and novel non-face objects showed face-specific congruency effects for young adults, but a loss of face-specificity in the congruency effects of older adults. This is a critical observation, since it was the specificity of the contextual interaction among attended and non-attend parts for faces or objects of expertise that let authors so far conclude a specific integrative processing mode from the observation of congruency effects (composite effects). The magnitude of congruency effects, as well as their association with response bias toward “different” responses for incongruent composites supports that a specific holistic processing mode is not lost at advanced ages. However, since, the congruency effect does also reflect part-based interference effects in older adults, as verified with non-face control objects, the face-congruency effect may confound both origins, part interference and holistic integration, and both sources can hardly be disentangled. It can therefore not be judged whether there is age-related decline in the face-specific component. We recommend not to ground conclusions about holistic face perception of older adults in a single measure, to combine several experimental paradigms which aim at different aspects of holistic processing, and to use non-face control objects to assess face-specificity.

## Ethics statement

The study was conducted in accordance with the Declaration of Helsinki. In detail, subjects participated voluntarily and gave written informed consent to their participation. In addition, participants were informed that they were free to stop the experiment at any time without negative consequences. The data were analyzed anonymously. All procedures, including personal treatment, data dandling and reasonability of experimental routines were approved by the local ethics committee at the Johannes Gutenberg University Mainz.

## Author contributions

All authors contributed equally to the conceptualization of the study. BM set up the basic design. MP conducted the experiments and data preparation. GM contributed data analysis and interpretation. All authors were involved in writing, preparation of the manuscript and final approval. All authors agree to be accountable for all aspects of the work in ensuring that questions related to the accuracy or integrity of any part of the work are investigated and resolved appropriately.

## Funding

This study was supported by the university research fund of Johannes Gutenberg University Mainz. Funding was granted to BM for project “Visual perception across the life-span.”

### Conflict of interest statement

The authors declare that the research was conducted in the absence of any commercial or financial relationships that could be construed as a potential conflict of interest.
